# Risk factors, physical and mental health burden of male and female pathological gamblers in the German general population aged 40–80

**DOI:** 10.1186/s12888-021-03110-8

**Published:** 2021-03-04

**Authors:** Martin Wejbera, Klaus Wölfling, Michael Dreier, Matthias Michal, Elmar Brähler, Jörg Wiltink, Andreas Schulz, Philipp S. Wild, Thomas Münzel, Jochem König, Karl Lackner, Norbert Pfeiffer, Manfred E. Beutel

**Affiliations:** 1 Department of Psychosomatic Medicine and Psychotherapy, University Medical Center of the Johannes Gutenberg University, Mainz, Germany; 2 Preventive Cardiology and Preventive Medicine - Center for Cardiology, University Medical Center of the Johannes Gutenberg University, Mainz, Germany; 3 Center for Thrombosis and Hemostasis, University Medical Center of the Johannes Gutenberg University, Mainz, Germany; 4 DZHK (German Center for Cardiovascular Research), Partner Site Rhine-Main, Mainz, Germany; 5 Center for Cardiology - Cardiology I, University Medical Center of the Johannes Gutenberg University, Mainz, Germany; 6 Institute of Medical Biostatistics, Epidemiology and Informatics, University Medical Center of the Johannes Gutenberg University, Mainz, Germany; 7 Institute of Clinical Chemistry and Laboratory Medicine, University Medical Center of the Johannes Gutenberg University, Mainz, Germany; 8 Department of Ophthalmology, University Medical Center of the Johannes Gutenberg University, Mainz, Germany

**Keywords:** Population survey, Gambling disorder, GD associations, GD prevalence, Migration background in GD, Gender differences in GD

## Abstract

**Background:**

Gambling Disorder (GD) has been associated with considerable mental and physical health risks in clinical samples. The paper determines risk factors, mental and physical health burden of probable GD for both men and women in the general population.

**Methods:**

In the Gutenberg Health Study, a population-based sample of *N* = 11,875 aged 40–80 years was analyzed regarding lifetime probable GD prevalence (measured with the Lie/ Bet Questionnaire) and a wide array of health variables including standardized measures of depression, anxiety, and somatic symptoms.

**Results:**

Probable GD lifetime prevalence was 2.1%, with higher rates among 1st generation migrants (5.5%; vs. non-migrants 1.6%), men (3.0%; vs. women 1.2%), and the sample’s youngest age decade (40–49 y., 3.1%). Lifetime probable GD was associated with current work-related, family and financial stressors as well as unhealthy behavior (smoking, extended screen time), and lifetime legal offenses. In men, but not in women, increased rates of imprisonment, mental and somatic symptoms were found.

**Conclusions:**

GD is a major public health problem with serious social, mental and physical health burden. Epidemiological findings underscore the preponderance of GD among 1st generation migrants and men. Findings are consistent with a vicious cycle of family, work related and financial stress factors, and mental and physical burden, particularly in men. Demographic risk factors may help to target specific prevention and treatment efforts.

**Supplementary Information:**

The online version contains supplementary material available at 10.1186/s12888-021-03110-8.

## Background

Banz and Lang [1] reported 12-month-prevalence rates between 0.31 and 0.82% for positive screens of gambling disorder plus 0.42 to 0.69% for positive screens of problematic gambling in their periodic surveys using the South Oaks Gambling Screen (SOGS [2]) in representative German samples. The PAGE study [3], the only German population survey to date that measured lifetime prevalence of gambling disorder (GD), found rates of 1.0% (CI 0.7–1.4%) for pathological gambling and an additional 1.4% (CI 1.1–1.8%) for problematic gambling according to DSM-IV criteria. Heterogeneous methodological aspects (e.g., assessment instrument, reference period) generally limit comparability of GD prevalence estimates [4], but the aforementioned surveys suggest that about 2 out of 100 Germans will exhibit problematic or pathological gambling during their lifetime and 1 out of 100 during the past 12 months.

Systematic reviews of international studies have identified male sex, younger age, being single or divorced, lower level of education, immigration, drug abuse, delinquency, depression, and anxiety as risk factors for GD [5, 6]. In German samples, male sex, young age, low educational level, and migration background (MB) were identified as risk factors [1, 3]. Kastirke et al. [7] confirmed MB as an independent risk factor for gambling problems while controlling for other demographic risk factors and gambling preference. 19.3 million people with MB were living in Germany in 2017, which equals 23.6% of the total population [8] and gives MB special importance in German GD research. The PAGE study [3] focused on comprehensive assessment of gambling behavior, GD criteria, and comorbidity, neglecting associations such as psychosocial and somatic risk factors and burden. The present study marks the first lifetime GD prevalence investigation based on a German general population sample that includes comprehensive assessments of psychosocial, somatic, and behavioral aspects, and the first to differentiate MB by first and second generation.

The association of GD and other mental health disorders, especially nicotine dependence and substance abuse, depression, and anxiety disorders, has been documented in a meta-analysis [9]. Morasco et al. [10] claimed to be the first to evaluate medical conditions among pathological gamblers and reported significant associations of lifetime GD diagnosis with tachycardia, angina pectoris, cirrhosis, and other liver disease. Higher rates of GD were found among coronary heart disease patients [11]. Pathological gamblers also reported more health-related concerns compared to non-gamblers [12], as well as more daily stress [13] and negative life events (among men [14], and among adolescents [15]). Numerous studies reported associations of gambling and crime [16]. Social support was a protective factor in relation to gambling problems [17, 18]. Systematic reviews of sex differences in GD reported greater psychological distress and unemployment in women, and higher rates of substance abuse among men [19, 20].

Based on a comprehensive data set from the only German major prospective cohort study to incorporate a screening for GD in addition to comprehensive assessments of somatic, behavioral and laboratory characterization to date, the purpose of the present paper was to answer the following questions: (1) what are psychosocial risk factors for GD, (2) does GD vulnerability differ between migration generations, (3) what is the mental and physical health burden of GD compared to participants without GD, and (4) do risk factors and health burden differ between male and female gamblers? We expected pathological gamblers to be more often male, younger, with MB, single/ divorced, and less educated. We further assumed a higher mental and physical health burden, more daily hassles and stressful life events (interpersonal conflicts, financial burdens, legal offenses).

## Methods

### Procedure and participants

The Gutenberg Health Study (GHS) at the University Medical Center Mainz in Germany is a population-based, prospective, observational single-center cohort study, with the primary aim to evaluate and improve cardiovascular risk stratification. Randomly selected from the registry office of Mainz city and its surrounding Mainz-Bingen district, the sample was stratified equally for gender, residence and decades of age. Potential participants with insufficient knowledge of German language or unable to visit the study center on their own (due to physical and/or mental impairment) were excluded. Baseline data of *N* = 15,010 aged 35 to 74 was collected from 2007 to 2012. The 5-h baseline-examination consisted of a computer-assisted personal interview and laboratory examinations in the study center that were conducted by certified medical technical assistants according to standard operating procedures. Wild et al. [21] have described the design and rationale of the GHS in detail.

From 2012 to 2017, the 5 year follow-up examinations were performed in the study center. With the exception of migration background, all data presented in this paper was derived from the follow-up examination, resulting in a cross-sectional analysis. Included were all participants who completed the Lie/ Bet Questionnaire (LBQ [22]), which was added at this time point. Cases with missing data in the LBQ (*n* = 548) were excluded from the analysis. The analyzed sample consisted of *N* = 11,875 respondents (48.8% female and 51.2% male). Age ranged from 40 to 80 years (mean = 59.2, SD 10.8).

### Measures

The Lie/ Bet Questionnaire (LBQ) is a 2-item screening instrument derived from the DSM-IV criteria for pathological gambling. It consists of two questions identified as the best predictors of pathological gambling (“Have you ever felt the need to bet more and more money?” and “Have you ever had to lie to people important to you about how much you gambled?”) with a dichotomous yes/no-answering format. Positive endorsement of one or both items is indicative of GD. Its sensitivity was 99%, its specificity 91% when distinguishing classified males with GD from male non-problem-gambling controls [22]. With a sensitivity of 100% and specificity of 85% in the follow-up study that also included female pathological gamblers and controls, it proved to be a useful screening device for a DSM based diagnosis of GD [23]. Adequate to detect problem gambling in mental health [24] and general populations [25], the instrument allows splitting the analyzed GHS sample into probable GD and no GD groups.

Migration background (MB) was defined according to the German micro-census of 2005 and registered in the initial baseline examination. It covers all individuals who migrated to the area of the Federal Republic of Germany after 1949 (1st generation migrants), and all non-German citizens born in Germany and all citizens born in Germany with at least one migrated parent or a parent abroad (2nd generation migrants). 22.5% of participants had a migration background, slightly exceeding official estimates of the MB proportion in Germany in 2015 with 21.1% [26]. We were able to form five 1st generation MB region groups of *n* > 100 based on geographic and cultural aspects (Eastern Europe, Western Europe/ America, Arabic-Islamic, Former Soviet Union, Former Yugoslavia; cf. supplemental material).

Current depression was assessed by the 9-question depression scale from the Patient Health Questionnaire (PHQ), the PHQ-9 [27], with a sum score of ≥10 defining caseness (range from 0 to 27; sensitivity of 88% and specificity of 88% for major depression). Current general anxiety disorder (GAD) was screened with the GAD-2, the short form (two items) of the GAD-7 [28], where a sum score of ≥3 (range 0–6) showed a sensitivity of 86% and a specificity of 83% for generalized anxiety [29]. The Mini-Social Phobia Inventory (Mini-Spin [30]) measured current social anxiety, with its cut-off score of 6 (range 0–12) reported to result in a sensitivity of 89% and a specificity of 90%. The Brief Social Support Scale (BS6 [31]) was used to assess current social support (range 6–24) and its two subscales tangible support and emotional-informational support (range 3–12 each), with higher scores indicating higher support. Current sleep disorder was assessed with the 4-item version of the Jenkins Sleep Scale (JSS-4 [32]) and its range from 0 to 20, where higher scores reflect greater sleeping problems (cut-off for disturbed sleep at 12). The PHQ-15 [33] was used to measure current somatic symptoms, where higher scores equate more somatic distress (item on menstruation was excluded, range from 0 to 28, cut-off for moderate somatization at 10). Stress was assessed with an adaptation of the Social Readjustment Rating Scale (SRRS [34]) consisting of 36 stress-inducing items dichotomized in 0 = *never occurred* and 1 = *occurred at any time in the past*. The dichotomized SRRS items “minor legal offense” and “imprisonment” were analyzed additionally due to their significance to GD. Furthermore, a list of 16 daily stressors (workplace and family-related items) composed for the GHS was used to measure current stress by focusing on the past 12 months, with response categories 1 = *did not occur*, 2 = *occurred but no strain*, 3 = *occurred and slight strain*, 4 = *occurred and moderate strain*, 5 = *occurred and severe strain*. The resulting scale ranged from 16 to 80, while the categories 1/ 2 were summarized to 0 = *no strain*, and 3/ 4/ 5 to 1 = *strain* for single item analysis of aspects expected to be associated with GD, namely financial burden, conflicts with superior/ colleagues/ within family, and loneliness (cf. [31]).

Other single item assessments include panic disorder (“In the last four weeks, have you had an anxiety attack—suddenly feeling fear or panic?” – yes/ no, with a sensitivity of 93% and a sensitivity of 78%, [35]), and screen time (“On an average weekday, how many hours of your spare time do you spend on a screen - like a TV, computer, other?” - more vs. less than 4 h per day). Current alcohol consumption was measured in gram per day (cutoff for abuse at 60 g/day for men and 40 g/day for women), Body Mass Index (BMI) in kg/m^2^, smoking status was dichotomized in non-smokers (never/ex-smokers) and smokers (occasional and frequent smokers).

Use of antidepressants/ anxiolytics, psychiatrist/ psychotherapist visited in the past 4 weeks, medical history of depression/ anxiety, and diseases (diabetes, obesity, COPD, hypertension, dyslipidemia, CVD, cancer, family history of myocardial infarction - MI/ stroke) were investigated during the follow-up visit (cf. [21]) and analyzed dichotomously (yes/no). Socioeconomic status (SES) was defined according to Lampert and Kroll [36], with scores ranging from 3 (lowest) to 21 (highest); this multi-dimensional index combines individual educational qualification with household characteristics of occupation and income with equal weights. Physical and mental health was self-rated on a scale from 1 = *very good* to 4 = *poor* each.

### Statistical analysis

As the population sample used in the GHS is stratified according to sex, age group, and urban versus suburban/rural origin, statistics describing the prevalence of probable GD were calculated by age group and weighted for the actual sex and age distribution of the 40–80 years old general population of Germany (as of 31.12.2015). Data are presented as numbers/ percentage or means with standard deviations. We performed Chi^2^-tests to compare probable GD and no GD groups. In order to identify associations of probable GD, we computed separate logistic regression models with probable GD as the dependent variable for the overall, male and female sample. The number of variables considered in the regression analysis had to be vastly reduced in order to obtain reliable results. Based on the literature, we selected sociodemographic (sex, age, SES, migration background), psychological (depression, GAD, panic disorder, daily stressors, social support, loneliness), behavioral (smoking, alcohol use, screen time), and somatic variables (BMI, somatic ailments). Models were pre-specified in a statistical analysis plan; interactions with sex were subsequently added. Complete case analysis was applied due to small proportions of missing values and large sample size. All *p*-values correspond to 2-tailed tests; the level of significance was set at *p* < .05. Statistical analysis was performed using the software R, version 3.5.1 [37].

## Results

### Prevalence

Two hundred thirty-five respondents were classified as individuals with probable GD according to the LBQ, of which 168 (71.5%) were male and 67 (28.5%) were female. Table 1 shows prevalence rates split for sex, age and MB. Probable GD rates were higher among men and first generation migrants, and within the youngest age decade (40–49 years old). 1st generation MB yielded a significantly higher percentage of probable GD for all regions, differences were most pronounced for Arabic-Islamic and former Yugoslavia regions (cf. supplemental Table 1).

**Table 1:** Probable Gambling Disorder prevalence rates (according to sex, migration background, and age)

		Men	Women
	% (95% CI)	% (95% CI)	% (95% CI)
Overall sample	2.1 [1.8; 2.3]	3.0 [2.6; 3.4]	1.2 [0.9; 1.5]
1st Gen. Migrants	5.5 [4.3; 7.1]	7.8 [5.8; 10.5]	3.4 [2.2; 5.4]
2nd Gen. Migrants	2.2 [1.6; 3.1]	2.4 [1.5; 3.8]	2.0 [1.2; 3.3]
Non-Migrants	1.6 [1.4; 1.9]	2.5 [2.1; 3.0]	0.7 [0.5; 1.1]
40–49 years	3.1 [2.5; 3.8]	4.5 [3.6; 5.7]	1.6 [1.1; 2.4]
50–59 years	2.1 [1.7; 2.6]	2.9 [2.2; 3.9]	1.2 [0.8; 1.9]
60–69 years	1.3 [0.9; 1.8]	1.9 [1.2; 2.8]	0.8 [0.4; 1.5]
70–79 years	1.4 [1.0; 2.0]	1.9 [1.2; 3.0]	1.0 [0.6; 1.8]

Presented are percentages and 95% confidence intervals

### Psychosocial characteristics

Table 2 shows psychosocial characteristics of the sample, and separately for men and women, comparing participants fulfilling criteria for probable GD (LBQ ≥ 1, referred to as “GD”) vs. those who do not (LBQ = 0, “no GD”).

**Table 2:** Psychosocial characteristics: Risk and protective factors for probable Gambling Disorder (GD) - men vs. women

Sample	Total sample			Men			Women		
Subgroup (n/%)	no GD (11,640/98.0%)	GD (235/2.0%)		no GD (5,912/97.2%)	GD (168/2.8%)		no GD (5,728/98.8%)	GD (67/1.2%)	
	% (n)	% (n)	p	% (n)	% (n)	p	% (n)	% (n)	p
DEMOGRAPHICS									
Age [years, mean (SD)]	59.3 (10.8)	56.0 (11.0)	*< 0.0001*	59.6 (10.8)	55.6 (10.9)	*< 0.0001*	59.0 (10.7)	57.1 (11.4)	0.17
SES [mean (SD)]	13.15 (4.39)	12.63 (4.61)	0.09	13.85 (4.38)	12.86 (4.79)	*0.009*	12.42 (4.28)	12.06 (4.09)	0.48
Education years [mean (SD)]	12.99 (2.05)	12.84 (2.37)	0.34	13.25 (2.10)	12.8 (2.36)	*0.018*	12.73 (1.97)	12.94 (2.41)	0.48
Partnership	85.0 (9862)	79.6 (187)	*0.027*	89.5 (5269)	81.0 (136)	*0.001*	80.4 (4593)	76.1 (51)	0.36
STRESS-RELATED									
Daily stressors (past 12 months)									
- conflicts with superior	9.8 (1121)	17.3 (40)	*0.0005*	9.9 (574)	16.9 (28)	*0.006*	9.7 (547)	18.5 (12)	*0.032*
- conflicts with colleagues	10.4 (1187)	17.0 (39)	*0.003*	10.8 (626)	16.4 (27)	*0.031*	10.0 (561)	18.5 (12)	*0.035*
- conflicts within family	24.1 (2765)	31.9 (74)	*0.008*	19.8 (1154)	31.3 (52)	*0.001*	28.6 (1611)	33.3 (22)	0.41
- financial burden	13.8 (1585)	33.9 (79)	*< 0.0001*	13.6 (792)	35.3 (59)	*< 0.0001*	14.1 (793)	30.3 (20)	*0.001*
- Total score [mean (SD)]	25.98 (7.77)	29.18 (9.25)	*< 0.0001*	25.37 (7.35)	29.04 (9.39)	*< 0.0001*	26.61 (8.14)	29.53 (8.93)	*0.01*
Life events (ever occurred)									
- minor legal offense	25.5 (2884)	42.3 (94)	*< 0.0001*	33.6 (1932)	45.0 (72)	*0.004*	17.1 (952)	35.5 (22)	*0.001*
- imprisonment	0.7 (83)	2.6 (6)	*0.009*	0.9 (54)	3.6 (6)	*0.006*	0.5 (29)	0 (0)	1.00
- Total score [mean (SD)]	16.59 (6.13)	17.31 (6.67)	0.10	16.14 (6.30)	17.14 (6.97)	0.07	17.05 (5.91)	17.72 (5.88)	0.36
SUPPORT [mean (SD)]									
Social support (total score)	9.76 (3.68)	10.74 (3.91)	*0.0002*	9.66 (3.60)	10.77 (4.03)	*0.001*	9.87 (3.75)	10.69 (3.64)	0.07
Social support (emotional)	5.04 (2.13)	5.69 (2.26)	*< 0.0001*	5.19 (2.15)	5.80 (2.34)	*0.001*	4.88 (2.09)	5.40 (2.03)	*0.041*
Social support (tangible)	4.74 (2.15)	5.08 (2.34)	*0.027*	4.49 (2.03)	5.00 (2.36)	*0.006*	5.00 (2.24)	5.28 (2.29)	0.31

The probable GD group indicated both a higher level of daily stress and more social support. Individual stressors (conflicts with superiors/ colleagues, financial burden) and stressful life events (minor legal offense, imprisonment) were more frequent. Individuals with probable GD were more often without an educational degree (11.5% vs. 5.5%, *p* = 0.0004; cf. supplemental Table 1).

Men with probable GD were younger, lived less frequently in partnerships (also less often married – 69.0% vs. 77.9%, *p* = 0.008 and more often divorced – 12.5% vs. 7.5%, *p* = 0.026), had lower SES, fewer education years and college degrees (9.0% vs. 16.8%, *p* = 0.006), and had more conflicts within family compared to men without GD. No such differences were found among women. Minor legal offenses were more frequent in both male and female probable GD groups, but imprisonment was more frequent in the male probable GD group only. Differences in social support were more pronounced among men.

### Mental and physical health burden

Table 3 presents the mental and physical health burden. Depression, generalized anxiety disorder, panic disorder, and social anxiety were considerably higher in the probable GD group – along with higher rates of history of depression and anxiety. The self-rating of mental health was worse within the probable GD group. When analyzed separately by sex, only men with probable GD reported more depression, generalized anxiety, panic disorder, history of depression, loneliness, and disturbed sleep, whereas only women with probable GD reported a higher history of anxiety disorders.

**Table 3:** Mental and physical health burden of probable Gambling Disorder (GD) - men vs. women

Sample	Total sample			Men			Women		
Subgroup (n/%)	no GD (11,640/98.0%)	GD (235/2.0%)		no GD (5912/97.2%)	GD (168/2.8%)		no GD (5728/98.8%)	GD (67/1.2%)	
	% (n)	% (n)	p	% (n)	% (n)	p	% (n)	% (n)	p
DISTRESS									
Depression (PHQ9 ≥ 10)	8.1 (937)	16.3 (38)	*< 0.0001*	6.3 (370)	16.8 (28)	*< 0.0001*	9.9 (567)	15.2 (10)	0.15
General Anxiety Disorder (GAD2 ≥ 3)	6.6 (759)	10.8 (25)	*0.016*	4.9 (288)	10.8 (18)	*0.002*	8.3 (471)	10.9 (7)	0.49
Panic Disorder	5.0 (575)	8.7 (20)	*0.021*	3.6 (212)	7.9 (13)	*0.010*	6.4 (363)	10.4 (7)	0.20
Social Anxiety	4.9 (565)	8.2 (19)	*0.031*	3.7 (217)	6.6 (11)	0.06	6.1 (348)	11.9 (8)	0.07
History of Depression	5.6 (654)	9.4 (22)	*0.022*	4.1 (244)	9.5 (16)	*0.003*	7.2 (410)	9.0 (6)	0.48
History of Anxiety Disorder	3.5 (407)	6.0 (14)	*0.049*	2.4 (142)	4.2 (7)	0.20	4.6 (265)	10.4 (7)	*0.037*
Loneliness	10.6 (1218)	14.8 (34)	0.051	8.4 (490)	15.8 (26)	*0.003*	12.9 (728)	12.3 (8)	1.00
Disturbed Sleep (JSS4 ≥ 12)	13.2 (1528)	17.0 (40)	0.10	10.0 (592)	16.7 (28)	*0.009*	16.4 (936)	17.9 (12)	0.74
Somatic Symptoms (PHQ15 ≥ 10)	17.2 (1988)	25.8 (60)	*0.001*	11.7 (687)	24.6 (41)	*< 0.0001*	22.9 (1301)	28.8 (19)	0.24
Mental health status [mean (SD)]	2.03 (0.64)	2.17 (0.70)	*0.003*	1.96 (0.61)	2.10 (0.69)	*0.008*	2.12 (0.65)	2.34 (0.69)	*0.009*
Physical health status [mean (SD)]	2.12 (0.60)	2.23 (0.63)	*0.008*	2.08 (0.59)	2.23 (0.63)	*0.002*	2.16 (0.61)	2.22 (0.65)	0.43
HEALTH BEHAVIOR									
Smoking (yes)	14.9 (1728)	28.5 (67)	*< 0.0001*	15.4 (907)	29.8 (50)	*< 0.0001*	14.4 (821)	25.4 (17)	*0.021*
Screen time (> 4 h)	17.2 (1932)	25.7 (58)	*0.001*	19.1 (1087)	25.0 (40)	0.07	15.3 (845)	27.3 (18)	*0.015*
Diabetes	10.3 (1198)	10.7 (25)	0.83	12.9 (760)	12.6 (21)	1.00	7.7 (438)	6.1 (4)	0.82
Obesity	25.8 (2997)	26.4 (62)	0.82	26.9 (1593)	25.0 (42)	0.66	24.5 (1404)	29.9 (20)	0.32
BMI [kg/m^2^, mean (SD)]	27.5 (5.0)	27.6 (5.2)	0.68	28.0 (4.4)	27.8 (5.0)	0.68	27.0 (5.6)	27.1 (5.6)	0.81
Alcohol abuse (> 60/40 g per day)	2.3 (273)	3.0 (7)	0.51	3.1 (185)	3.6 (6)	0.65	1.5 (88)	1.5 (1)	1.00
SOMATIC DISEASES									
COPD	4.8 (559)	8.1 (19)	*0.031*	4.1 (240)	8.3 (14)	*0.016*	5.6 (319)	7.5 (5)	0.42
CVD	14.1 (1631)	15.4 (36)	0.57	17.7 (1039)	17.3 (29)	1.00	10.4 (592)	10.6 (7)	0.84
Cancer	10.7 (1239)	10.2 (24)	0.92	10.5 (619)	8.3 (14)	0.44	10.8 (620)	14.9 (10)	0.32

Regarding physical health and health behavior, almost twice the proportion of the probable GD group were smokers. A daily screen time of > 4 h, which can be taken as indicative of sedentary life style, and somatic symptoms were reported more often within the probable GD group, while self-rated physical health was worse. Of the somatic diseases, only COPD rate was increased in the probable GD group, but neither CVD, cancer, nor other variables indicative of physical health. COPD and somatic symptoms were more frequent, and self-rated physical health was worse in the male, but not the female probable GD group, while only women with probable GD reported a daily screen time of > 4 h significantly more often.

### Regression analysis

In multivariable analysis (Table 4), the strongest predictors of probable GD were 1st generation MB (OR = 3.12) and male sex (OR = 2.90). A significantly higher likelihood of probable GD was also found for smoking (OR = 1.79), high screen time (OR = 1.48), daily stressors (OR = 1.18) and somatic symptoms (O = 1.04) as well as lower SES (OR = 0.95) and lower age (0.98).

**Table 4:** Prediction of Gambling Disorder (GD) by sociodemographic, psychological, behavioral, and somatic variables

	Total sample (***n*** = 11,089)			Men (***n*** = 5706)			Women (***n*** = 5383)		
Variable	OR	95%CI	*p*	OR	95%CI	*p*	OR	95%CI	*p*
Sex (Men)	2.90	2.13–3.99	*< 0.0001*						
Age [y]	0.98	0.96–0.99	*0.005*	0.97	0.95–0.99	*0.001*	1.00	0.97–1.03	0.99
SES	0.95	0.92–0.99	*0.008*	0.95	0.91–0.99	*0.008*	0.99	0.92–1.06	0.73
1st Gen. Migrants (vs non)	3.12	2.22–4.34	*< 0.0001*	2.71	1.79–4.02	*< 0.0001*	4.36	2.32–7.92	*< 0.0001*
2nd Gen. Migrants (vs non)	1.15	0.74–1.72	0.52	0.77	0.42–1.32	0.37	2.35	1.18–4.42	*0.010*
Depression (PHQ9 ≥ 10)	1.20	0.71–1.98	0.50	1.28	0.68–2.35	0.44	1.02	0.38–2.52	0.97
General Anxiety Disorder (GAD2 ≥ 3)	0.76	0.43–1.32	0.35	0.77	0.38–1.50	0.46	0.79	0.27–2.09	0.65
Panic disorder	1.29	0.73–2.17	0.36	1.19	0.57–2.29	0.63	1.54	0.59–3.50	0.34
Daily stressors^a^ [per 5 points]	1.18	1.07–1.29	*0.001*	1.17	1.04–1.30	*0.009*	1.21	1.02–1.43	*0.028*
Social support (total)^a^ [per 1 point]	1.02	0.98–1.06	0.28	1.02	0.97–1.07	0.42	1.03	0.95–1.10	0.47
Loneliness	0.78	0.49–1.20	0.27	0.9	0.53–1.50	0.70	0.52	0.20–1.16	0.13
Smoking	1.79	1.30–2.44	*0.0003*	1.78	1.22–2.56	*0.002*	1.85	0.98–3.31	*0.046*
Alcohol abuse	0.86	0.30–1.92	0.74	0.83	0.25–2.04	0.72	1.06	0.06–5.06	0.96
Screen time (> 4 h)	1.48	1.06–2.02	*0.017*	1.33	0.90–1.94	0.14	1.92	1.04–3.38	*0.029*
BMI [kg/m^2^]	0.98	0.95–1.01	0.20	0.97	0.93–1.00	0.08	1	0.96–1.05	0.88
Somatic symptoms^**a**^ [per 1 point]	1.04	1.00–1.08	*0.035*	1.06	1.01–1.11	*0.017*	1.01	0.94–1.08	0.75

Dependent variable: GD. *OR* Odds Ratio, *95%CI* 95% Confidence Interval

^a^ *scaled variables (Daily stressors from 16 to 80 / Social Support from 6 to 24 / Somatic symptoms from 0 to 28)*

When analyzed separately for men and for women, SES and age were only significant for men (OR = 0.95 and OR = 0.97, respectively), and so were somatic symptoms (OR = 1.06). The risk of probable GD was increased in 2nd generation migrants (OR = 2.35) and with high screen times (OR = 1.92) exclusively for women. The interaction term for sex and 2nd gen. MB is significant, while all other interaction terms for sex and the aforementioned variables are not (with interaction term for sex and age close to significance, cf. supplemental Table 2).

All 1st generation migrants’ regions of origin increased the likelihood of probable GD significantly. Strongest predictors among regions of origin were Arabic-Islamic countries (OR = 5.50) and former Yugoslavia (OR = 4.42), but former Soviet Union (OR = 2.90), Western Europe/ America (OR = 2.46) and Eastern Europe (OR = 2.14) were also significant (cf. supplemental Table 3).

## Discussion

To our knowledge, the present study is the only major prospective cohort study to date performing comprehensive assessments of somatic, behavioral and laboratory characterization while incorporating a GD screener. It demonstrated a considerable rate of probable lifetime GD with major adverse consequences. It aimed primarily to identify risk factors of lifetime GD and the influence of MB generation as well as MB region. *N* = 235 cases with probable GD corresponded to a lifetime prevalence rate of 2.1% and allowed for comparisons between men (*n* = 168) and women (*n* = 67) with vs. without GD. 1st generation MB and male sex emerged as the strongest predictors, tripling the likelihood of probable GD, while 2nd generation MB was significant among women only. Migration from Arabic-Islamic countries and former Yugoslavia was most strongly associated with lifetime probable GD among regions investigated. This is in line with previous findings, as Kastirke et al. [38] identified mostly Turkish, but also Yugoslavian and to a lesser degree Asian MB as significant risk factors in the German population. The “immigrant paradox” suggested for GD in the United States (where subsequent generations of immigrants had higher gambling and GD prevalence than 1st generation immigrants [39]) seems not applicable to Germany, as our differentiation of MB generations showed a higher probable GD risk for 1st generation migrants.

Probable GD was consistently associated with increased daily stressors (conflicts with superior, colleagues, financial burden) and smoking, furthermore higher rates of (lifetime and current) depression and anxiety, disturbed sleep, an increased rate of minor legal offenses, high screen time, somatic symptoms, and lower self-rated physical health. Based on our findings, we would postulate a vicious cycle of family, work related and financial stress factors, unhealthy behavior, and mental as well as physical burden. Social support has been demonstrated as a protective factor in GD development, but was increased in our probable GD group. As we inquired about lifetime probable GD, we cannot differentiate if some of our participants have overcome GD by means of social support. On the other hand, reliance on social support may be a double-edged sword for pathological gamblers, who are often known to overburden their support system and generate codependency (by gambling more than their expendable income and then relying on social support, e.g. stealing/borrowing in excess of their support system’s capacity, to get by and/or finance further gambling behavior).

Our findings emphasize the need to discriminate risk factors and burdens of GD by sex. 2nd generation MB was a significant predictor of probable GD for women only. Men with probable GD were faced with more challenges in maintaining relationships (conflicts/ divorce/ unmarried/ no partnership/ loneliness), whereas no such differences were found for women. More sex-specific negative outcomes were observed on somatic variables for men (more COPD, somatic symptoms, disturbed sleep, self-rated physical health), and on the behavioral level for women (higher screen time).

GD studies used to focus on men because they were predominant in the addiction treatment/ health care system. Reported male proportions among current GD treatment seekers in Germany ranged from 82.4% for inpatient [40] to 89.3% for outpatient treatment [41], while sex-ratio for GD in population samples were not as uneven (2.5:1 in our sample; 2.2:1 in [1]). Three reasons may apply: (1) Gambling among women may be less socially accepted, leading to lower readiness to acknowledge GD and seek treatment; (2) Men may suffer from more severe GD pathology. The prevalence rates of Banz and Lang [1] can be split into 0.55% pathological plus 0.64% problematic gamblers for men, and 0.06% pathological plus 0.47% problematic gamblers for women, indicating a lower degree of problem severity in women; (3) Earlier pathological gambling onset has been observed in men [19, 42, 43], and Black et al. [44] reported higher rates of co-occurring mental health and addictive disorders in early onset-cases. It can be surmised that our male probable GD group exhibited greater severity as well, based on their more pronounced social and health burdens compared to women. As the LBQ gives no information about the extent of disorder, more conclusive evidence is needed to explain the gap between treatment seeking and epidemiological data.

### Strengths and limitations

We performed a comprehensive evaluation of lifetime probable GD and mental and physical health factors, in a German general population sample that is the first to distinguish MB by first and second generation. Demographic findings are relevant as risk factors for GD prevention and therapy; however, it is not possible to further differentiate causes and consequences based on cross-sectional data.

The LBQ’s higher negative prediction value [23] means that the reported lifetime prevalence of 2.1% is more likely to over- than underestimate probable GD among 40 to 80 year-olds in Germany, as false positives are more likely than false negatives. On the other hand, lifetime (as well as past year) GD has been more commonly reported by younger vs. older adults [3, 5]. Thus, lifetime GD prevalence within the total adult population may be higher than estimated based on our sample. Further research with the LBQ in a German sample including 18 to 39 year-olds, who were not recruited in this sample due to the GHS focus on cardiovascular risk population, could give conclusive answers.

GD has been shown to be not uniformly distributed geographically when residence district deprivation is considered, with moderate but significant area effects reported in UK, and similar findings from North America and Australia [45]. This mechanism has not been studied in Germany as of today and may limit the extrapolation of prevalence findings in our sample to the general population.

The paper was based on self-report data, which are subject to known fallacies (reliability of memory, social desirability). Negative connotations of gambling behavior and the very nature of GD itself (hiding/ lying about the extent of ones gambling behavior) may have led to socially desirable answers to the LBQ. Our research did not cover current or recent prevalence of gambling problems, as the used LBQ refers to lifetime occurrence. A more extensive evaluation of GD criteria would have been desirable, but was not possible due to limited interview duration.

## Conclusions

Findings suggest multiple pathways of psychosocial, mental and physical health damage of GD and alert to risk groups. Overall, MB and male sex proved to be the disorder’s best predictors. With almost a quarter of the German population having MB, their GD vulnerability is a relevant social and health risk; however, except for occasional Turkish and Russian offers, counselling/ treatment options and prevention material are usually available in German only. More treatment and prevention measures need to be targeted specifically at this risk group, considering linguistic and/ or cultural challenges. 2nd generation MB was a specific risk factor for women. Younger age, lower SES and somatic burden were found to be of higher relevance to probable GD among men, as were high screen times among women. Sex differences regarding GD associations on somatic/ behavioral levels should be investigated further and incorporated in preventive approaches. Further including women in GD research may enable a better understanding of the disorder and their lower treatment-seeking behavior.

## Supplementary Information

**Additional file 1.**

## Data Availability

For approved reasons, some access restrictions apply to the data underlying these findings. Data sets contain identifying participant information, which is not suitable for public deposition. Access to the local database is available upon request to the corresponding author.

## Abbreviations

GD: Gambling disorder
GAD: General anxiety disorder
GHS: Gutenberg health study
LBQ: Lie/ bet questionnaire
MB: Migration background
