# Supplementary material for: Risk factors, physical and mental health burden of male and female pathological gamblers in the German general population aged 40–80
Source: BMC Psychiatry. 2021 Mar 4;21:123. doi: 10.1186/s12888-021-03110-8 (PMC7931586; doi:10.1186/s12888-021-03110-8)
Supplement: Supplementary file 1 — Additional file 1. [file 12888_2021_3110_MOESM1_ESM.docx]

Supplemental Table 1. Other risk and protective factors for probable Gambling Disorder (GD) - men vs. women

| **Sample** | **Total sample** | | | **Men** | | | **Women** | | |
| --- | --- | --- | --- | --- | --- | --- | --- | --- | --- |
| Subgroup (n) | no GD (11,640) | GD (235) |  | no GD (5,912) | GD (168) |  | no GD (5,728) | GD (67) |  |
|  | % (n) | % (n) | p | % (n) | % (n) | p | % (n) | % (n) | p |
| Living with partner | 88.5 (9316) | 85.9 (176) | 0.27 | 90.7 (4993) | 86.6 (129) | 0.09 | 86.0 (4323) | 83.9 (47) | 0.70 |
| Household size | 2.34 (1.03) | 2.46 (1.28) | 0.16 | 2.41 (1.03) | 2.59 (1.41) | 0.10 | 2.27 (1.04) | 2.12 (0.81) | 0.14 |
| employment | 57.4 (6659) | 66.4 (156) | *0.006* | 59.8 (3518) | 69.6 (117) | *0.010* | 55.0 (3141) | 58.2 (39) | 0.62 |
| fulltime employment | 41.0 (4750) | 54.9 (129) | *<0.0001* | 54.1 (3182) | 63.1 (106) | *0.023* | 27.5 (1568) | 34.3 (23) | 0.22 |
| Income (individ.) [€/mo; mean (SD)] | 2330.2 (2193.6) | 2471.2 (2656.5) | 0.43 | 3061.7 (2515.0) | 2819.1 (2961.6) | 0.31 | 1527.6 (1385.6) | 1567.7 (1236.5) | 0.80 |
| Income (househ.) [€/mo; mean (SD)] | 3792.7 (2720.8) | 3722.5 (3169.0) | 0.74 | 4031.6 (2834.2) | 3763.9 (3116.3) | 0.29 | 3535.4 (2568.8) | 3616.9 (3323.1) | 0.85 |
|  |  |  |  |  |  |  |  |  |  |
| Western Europe/ America MB | 1.7 (193) | 3.8 (9) | *0.019* | 1.6 (94) | 3.6 (6) | 0.058 | 1.7 (99) | 4.5 (3) | 0.11 |
| Former Soviet Union MB | 1.3 (148) | 3.4 (8) | *0.012* | 1.0 (62) | 2.4 (4) | 0.11 | 1.5 (86) | 6.0 (4) | *0.020* |
| Former Yugoslavia MB | 0.8 (97) | 3.0 (7) | *0.005* | 0.8 (45) | 3.6 (6) | *0.003* | 0.9 (52) | 1.5 (1) | 0.46 |
| Eastern Europe MB | 2.7 (316) | 5.5 (13) | *0.015* | 2.6 (151) | 4.8 (8) | *0.084* | 2.9 (165) | 7.5 (5) | *0.046* |
| Arabic-Islamic MB | 1.2 (137) | 7.2 (17) | *<0.0001* | 1.6 (92) | 7.7 (13) | *<0.0001* | 0.8 (45) | 6.0 (4) | *0.002* |
|  |  |  |  |  |  |  |  |  |  |
| Marital status |  |  |  |  |  |  |  |  |  |
| - single | 9.2 (1068) | 11.9 (28) | 0.17 | 10.0 (588) | 12.5 (21) | 0.30 | 8.4 (480) | 10.4 (7) | 0.51 |
| - married | 74.2 (8622) | 67.7 (159) | *0.029* | 77.9 (4598) | 69.0 (116) | *0.008* | 70.4 (4024) | 64.2 (43) | 0.28 |
| - civil union | 0.1 (17) | 0.4 (1) | 0.30 | 0.2 (11) | 0.6 (1) | 0.29 | 0.1 (6) | 0 (0) | 1.00 |
| - divorced | 9.0 (1043) | 11.1 (26) | 0.25 | 7.5 (444) | 12.5 (21) | *0.026* | 10.5 (599) | 7.5 (5) | 0.55 |
| - separated | 1.6 (190) | 3.0 (7) | 0.12 | 1.6 (96) | 3.0 (5) | 0.20 | 1.6 (94) | 3.0 (2) | 0.31 |
| - widowed | 5.8 (678) | 6.0 (14) | 0.89 | 2.8 (164) | 2.4 (4) | 1.00 | 9.0 (514) | 14.9 (10) | 0.13 |
|  |  |  |  |  |  |  |  |  |  |
| Education (highest) |  |  |  |  |  |  |  |  |  |
| - no degree | 5.5 (634) | 11.5 (27) | *0.0004* | 2.7 (159) | 9.6 (16) | *<0.0001* | 8.3 (475) | 16.4 (11) | *0.026* |
| - lowest secondary | 34.2 (3964) | 32.1 (75) | 0.53 | 34.3 (2020) | 35.3 (59) | 0.80 | 34.0 (1944) | 23.9 (16) | 0.091 |
| - secondary | 25.1 (2907) | 21.4 (50) | 0.22 | 19.3 (1135) | 16.8 (28) | 0.49 | 31.0 (1772) | 32.8 (22) | 0.79 |
| - highest secondary | 10.3 (1196) | 13.2 (31) | 0.16 | 13.6 (801) | 13.2 (22) | 1.00 | 6.9 (395) | 13.4 (9) | 0.050 |
| - apprenticeship | 45.8 (5313) | 43.6 (102) | 0.51 | 37.5 (2206) | 44.3 (74) | 0.08 | 54.4 (3107) | 41.8 (28) | *0.048* |
| - master craftsman | 15.6 (1813) | 15.0 (35) | 0.86 | 19.4 (1141) | 15.0 (25) | 0.16 | 11.8 (672) | 14.9 (10) | 0.44 |
| - college degree | 12.3 (1432) | 9.4 (22) | 0.19 | 16.8 (989) | 9.0 (15) | *0.006* | 7.8 (443) | 10.4 (7) | 0.36 |
|  |  |  |  |  |  |  |  |  |  |
| Visited psychiatrist/ psychotherapist | 0.4 (51) | 0.4 (1) | 1.00 | 0.2 (12) | 0 (0) | 1.00 | 0.7 (39) | 1.5 (1) | 0.37 |
| Antidepressant intake (current) | 6.1 (706) | 5.5 (13) | 0.89 | 3.9 (228) | 4.2 (7) | 0.84 | 8.4 (478) | 9.0 (6) | 0.82 |
| Anxiolytic intake (current) | 0.9 (110) | 0.9 (2) | 1.00 | 0.6 (35) | 1.2 (2) | 0.27 | 1.3 (75) | 0 (0) | 1.00 |
| GAD2 score [mean (SD)] | 0.95 (1.09) | 1.26 (1.21) | *0.0001* | 0.79 (1.01) | 1.17 (1.23) | *<0.0001* | 1.11 (1.14) | 1.52 (1.15) | *0.007* |
| PHQ9 score [mean (SD)] | 4.27 (3.60) | 5.83 (4.35) | *<0.0001* | 3.76 (3.41) | 5.71 (4.30) | *<0.0001* | 4.8 (3.71) | 6.13 (4.52) | *0.004* |
| PHQ15 score [mean (SD)] | 5.94 (4.00) | 7.07 (4.58) | *0.0002* | 5.12 (3.66) | 6.82 (4.74) | *<0.0001* | 6.79 (4.16) | 7.71 (4.12) | 0.07 |
| Qualitiy of sleep [JSS-4; mean (SD)] | 5.40 (4.79) | 6.23 (5.18) | *0.016* | 4.76 (4.47) | 5.91 (5.09) | *0.004* | 6.06 (5.01) | 7.01 (5.35) | 0.15 |
| Mental health status [mean (SD)] | 2.03 (0.64) | 2.17 (0.70) | *0.003* | 1.96 (0.61) | 2.10 (0.69) | *0.008* | 2.12 (0.65) | 2.34 (0.69) | *0.009* |
| Physical health status [mean (SD)] | 2.12 (0.60) | 2.23 (0.63) | *0.008* | 2.08 (0.59) | 2.23 (0.63) | *0.002* | 2.16 (0.61) | 2.22 (0.65) | 0.43 |
| Hypertension (yes) | 53.5 (6217) | 46.4 (109) | *0.034* | 58.7 (3462) | 46.4 (78) | *0.002* | 48.2 (2755) | 46.3 (31) | 0.81 |
| Dyslipidemia (yes) | 34.3 (3982) | 35.0 (82) | 0.83 | 41.4 (2439) | 41.1 (69) | 1.00 | 27.0 (1543) | 19.7 (13) | 0.21 |
| FH of MI/ Stroke (yes) | 23.3 (2716) | 27.2 (64) | 0.16 | 21.6 (1278) | 25.0 (42) | 0.30 | 25.1 (1438) | 32.8 (22) | 0.16 |

Supplemental Table 2. Prediction of probable Gambling Disorder (GD) by sociodemographic, psychological, behavioral, and somatic variables; interaction terms included

|  | **Total sample (n=11,089)** | | |  |
| --- | --- | --- | --- | --- |
| Variable | OR | 95%CI | p |  |
| Sex (Men) | 3.40 | 2.18 - 5.47 | *<0.0001* |  |
| Age [y] | 1.00 | 0.97 - 1.03 | 0.97 |  |
| Sex (Men) × Age | 0.97 | 0.94 - 1.00 | 0.058 |  |
| SES | 0.98 | 0.92 - 1.05 | 0.64 |  |
| Sex (Men) × SES | 0.96 | 0.89 - 1.04 | 0.32 |  |
| 1st Gen. Migrants (vs non) | 4.31 | 2.31 - 7.80 | *<0.0001* |  |
| 2nd Gen. Migrants (vs non) | 2.33 | 1.17 - 4.37 | *0.011* |  |
| Sex (Men) × 1st Gen. Migrants | 0.63 | 0.31 - 1.32 | 0.22 |  |
| Sex (Men) × 2nd Gen. Migrants | 0.33 | 0.14 - 0.80 | *0.013* |  |
| Depression (PHQ9≥10) | 1.20 | 0.71 - 1.99 | 0.49 |  |
| General Anxiety Disorder (GAD2≥3) | 0.77 | 0.43 - 1.34 | 0.37 |  |
| Panic disorder | 1.31 | 0.74 - 2.20 | 0.33 |  |
| Daily stressors [per 5 points] | 1.18 | 1.07 - 1.29 | *0.001* |  |
| Social support (total) [per 1 point] | 1.02 | 0.98 - 1.06 | 0.28 |  |
| Loneliness | 0.78 | 0.49 - 1.19 | 0.26 |  |
| Smoking | 1.79 | 1.30 - 2.44 | *0.0003* |  |
| Alcohol abuse | 0.86 | 0.30 - 1.95 | 0.75 |  |
| Screen time (>4h) | 1.95 | 1.06 - 3.42 | *0.024* |  |
| Sex (Men) × Screen time | 0.68 | 0.34 - 1.38 | 0.27 |  |
| BMI [kg/m²] | 0.98 | 0.95 - 1.01 | 0.19 |  |
| Somatic symptoms [per 1 point] | 1.01 | 0.95 - 1.07 | 0.73 |  |
| Sex (Men) × Somatic symptoms | 1.05 | 0.98 - 1.12 | 0.17 |  |

Dependent variable: GD. OR = Odds Ratio, 95%CI = 95% Confidence Interval

Supplemental Table 3. Prediction of probable Gambling Disorder (GD) by sociodemographic, psychological, behavioral, and somatic variables; 1^st^ Gen. MB region specified

|  | **Total sample (n=9,612)** | | |
| --- | --- | --- | --- |
| Variable | OR | 95%CI | p |
| Sex (Men) | 3.20 | 2.28 - 4.56 | *<0.0001* |
| Age [y] | 0.98 | 0.96 - 0.99 | *0.007* |
| SES | 0.95 | 0.92 - 0.99 | *0.009* |
| 1st Gen. MB. Eastern Europe (vs all other) | 2.14 | 1.07 - 3.88 | *0.020* |
| 1st Gen. MB. Western Europe-America (vs all other) | 2.46 | 1.08 - 4.87 | *0.017* |
| 1st Gen. MB. former Soviet Union (vs all other) | 2.90 | 1.25 - 5.87 | *0.006* |
| 1st Gen. MB. Arabic-Islamic (vs all other) | 5.50 | 2.97 - 9.63 | *<0.0001* |
| 1st Gen. MB. former Yugoslavia (vs all other) | 4.42 | 1.78 - 9.40 | *0.0004* |
| Depression (PHQ9≥10) | 1.03 | 0.58 - 1.79 | 0.92 |
| General Anxiety Disorder (GAD2≥3) | 0.76 | 0.40 - 1.37 | 0.38 |
| Panic disorder | 1.06 | 0.55 - 1.92 | 0.86 |
| Daily stressors [per 5 points] | 1.16 | 1.05 - 1.28 | *0.004* |
| Social support (total) [per 1 point] | 1.03 | 0.99 - 1.08 | 0.10 |
| Loneliness | 0.87 | 0.54 - 1.37 | 0.56 |
| Smoking | 1.81 | 1.29 - 2.51 | *0.0005* |
| Alcohol abuse | 0.80 | 0.24 - 1.95 | 0.66 |
| Screen time (>4h) | 1.72 | 1.22 - 2.39 | *0.002* |
| BMI [kg/m²] | 0.98 | 0.95 - 1.01 | 0.20 |
| Somatic symptoms [per 1 point] | 1.04 | 1.00 - 1.09 | 0.051 |

Dependent variable: GD. OR = Odds Ratio, 95%CI = 95% Confidence Interval

1st generation Migration Background - regions

Eastern Europe (329) – Bulgaria, Czechoslovakia (today: Czech Republic, Slovakia), Hungary, Poland, Romania

Western Europe/America (202) – Belgium, Denmark, Finland, France, Great Britain, Greece, Ireland, Italy, Luxembourg, Malta, Netherlands, Norway, Portugal, Spain, Sweden, Switzerland, Canada, USA, Australia

Former Soviet Union (156) – Armenia, Azerbaijan, Belarus, Estonia, Georgia, Kazakhstan, Kirghizia, Kyrgyzstan, Latvia, Lithuania, Moldova, Russia, Tadzhikistan, Turkmenistan, Ukraine, Uzbekistan

Arabic-Islamic (154) – Algeria, Afghanistan, Bangladesh, Egypt, Ghana, Guinea-Bissau, Indonesia, Iran, Iraq, Lebanon, Morocco, Pakistan, Senegal, Syria, Tunisia, Turkey

Former Yugoslavia (104) – Bosnia-Herzegovina, Croatia, Kosovo, Macedonia, Serbia and Montenegro, Slovenia, Yugoslavia
